# Electrical Properties of Ultrathin Hf-Ti-O Higher *k* Gate Dielectric Films and Their Application in ETSOI MOSFET

**DOI:** 10.1186/s11671-016-1754-5

**Published:** 2016-11-30

**Authors:** Yuhua Xiong, Xiaoqiang Chen, Feng Wei, Jun Du, Hongbin Zhao, Zhaoyun Tang, Bo Tang, Wenwu Wang, Jiang Yan

**Affiliations:** 1 Advanced Electronic Materials Institute, General Research Institute for Nonferrous Metals, Beijing, 100088 China; 2 Key Laboratory of Microelectronics Devices and Integrated Technology, Institute of Microelectronics, Chinese Academy of Sciences, Beijing, 100029 China

**Keywords:** Ultrathin Hf-Ti-O gate dielectric films, Higher k, Atomic layer deposition, Electrical properties, ETSOI MOSFET

## Abstract

Ultrathin Hf-Ti-O higher *k* gate dielectric films (~2.55 nm) have been prepared by atomic layer deposition. Their electrical properties and application in ETSOI (fully depleted extremely thin SOI) PMOSFETs were studied. It is found that at the Ti concentration of Ti/(Ti + Hf) ~9.4%, low equivalent gate oxide thickness (EOT) of ~0.69 nm and acceptable gate leakage current density of 0.61 A/cm^2^ @ (*V*_fb_ − 1)*V* could be obtained. The conduction mechanism through the gate dielectric is dominated by the F-N tunneling in the gate voltage range of −0.5 to −2 V. Under the same physical thickness and process flow, lower EOT and higher *I*_on_/*I*_off_ ratio could be obtained while using Hf-Ti-O as gate dielectric compared with HfO_2_. With Hf-Ti-O as gate dielectric, two ETSOI PMOSFETs with gate width/gate length (*W*/*L*) of 0.5 μm/25 nm and 3 μm/40 nm show good performances such as high *I*_on_, *I*_on_/*I*_off_ ratio in the magnitude of 10^5^, and peak transconductance, as well as suitable threshold voltage (−0.3~−0.2 V). Particularly, ETSOI PMOSFETs show superior short-channel control capacity with DIBL <82 mV/*V* and subthreshold swing <70 mV/decade.

## Background

On the basis of International Technology Roadmap for Semiconductors (ITRS) 2013 [1], reduction of the equivalent gate oxide thickness (EOT) below 0.7 nm with appropriate metal gates remains as the most difficult challenge associated with the future device scaling.

Hf-based oxide high-*k* has been applied in 45- [2], 32-, 22-, and 14-nm technology nodes. An apparent way to scale EOT is to reduce the physical thickness of the Hf-based oxide. However, there is little room in this direction. One of the possible EOT scaling approaches is to introduce a new high-*k* material with *k* value greater than that of HfO_2_ [3, 4], particularly higher *k* (*k* > 30) [1].

Considering the process compatibility of Hf-based oxide high-*k*, investigation on the electrical properties of Hf-based higher *k* gate dielectrics is of significance in extending Hf-based high-*k* to the future nodes as well as continuing CMOS scaling. One way to increase the permittivity of HfO_2_ is combining it with very high-*k* materials, for instance TiO_2_ with a *k* value of 50~80 due to remote phonon scattering [5, 6]. Introducing Ti into HfO_2_ could tune the *k* value according to the Ti content, thus achieving desired *k* value [7, 8]. Ultrathin EOT (~8 Å) was achieved by using bi-layer sputtered TiO_2_/HfO_2_ dielectric with effective permittivity ~36 [9].

Recently, as the mainstream bulk devices face formidable challenges to scale beyond 20-nm node, there is an increasingly renewed interest in fully depleted devices such as FinFET and ETSOI for continued CMOS scaling [10]. ETSOI MOSFET is considered as one of the main options for continued MOSFET scaling in 22- and 16/14-nm technology nodes, owing to its superior short-channel control capacity and immunity to random dopant fluctuation [11–14].

The previous studies have rarely utilized Hf-Ti-O higher *k* in short-channel MOSFET especially ETSOI MOSFET to investigate the effect of Hf-Ti-O on device performances including *I*_on_/*I*_off_ ratio (switch ratio) and short-channel effects. Investigation on the application of Hf-Ti-O higher *k* in ETSOI MOSFET, a new device structure will help to evaluate practicability of Hf-Ti-O in the future technology nodes and continue CMOS scaling.

In this study, in order to obtain EOT below 0.7 nm, ultrathin Hf-Ti-O higher *k* gate dielectric films (~2.55 nm) have been prepared by atomic layer deposition (ALD). Their electrical properties and application in short-channel ETSOI PMOSFETs were studied. For contrast, MOS capacitor and ETSOI MOSFET with HfO_2_ (~2.55 nm) as high-*k* gate dielectric were prepared as control samples.

## Methods

### Preparation of the Hf-Ti-O Higher *k* and MOS Capacitors

The MOS capacitors were prepared on 8-in. p-Si (100) substrates with a resistivity of 8~12 Ω cm. Since high *k*/Si interface quality is critical to the EOT scaling and device performance, ~0.6-nm SiO_2_ interfacial layer (IL) was intentionally grown by ozone oxidization of Si before Hf-Ti-O higher *k* deposition. [(CH_3_)(C_2_H_5_)N]_4_Hf and TiCl_4_ were used as the metal precursors. Deionized water was chosen as an oxygen source and N_2_ (99.999%) as a carrier and purge gas. The substrate temperature was kept at 300 °C. Sixteen-cycle HfO_2_/4-cycle TiO_2_/16-cycle HfO_2_ sandwich structure was utilized to reduce the Ti diffusion. As for the control sample, 34-cycle HfO_2_ was prepared. Then, PDA (post deposition annealing) in 90% N_2_/10% O_2_ at 450 °C for 15 s was performed. Then, the TiN was used as the metal gate with a gate area of 100 × 100 μm^2^ and *W* as the capping layer. After gate patterning, backside Al was deposited for the ohmic contact and the forming gas annealing was carried out in 95% N_2_/5% H_2_ at 450 °C for 20 min.

### Characterization of the Hf-Ti-O Higher k/IL/Si Stack and Electrical Properties

The gate stack structure was characterized by high-resolution transmission electron microscopy (HRTEM). The Hf-Ti-O film composition and interfacial reaction were investigated by XPS. High-frequency capacitance–voltage (*C*–*V*) at 1 MHz and gate leakage current density-gate voltage (*J*_*g*_–*V*_*g*_) measurements were performed for the MOS capacitors. The EOT and flat-band voltage (*V*_fb_) were extracted by fitting the measured high frequency *C*–*V* data through a *C*–*V* simulator developed by UC Berkeley, including quantum mechanical effect.

### Preparation of the ETSOI PMOSFETs

The ETSOI PMOSFETs were fabricated on 8-in. SOI wafers with a buried oxide (BOX) thickness of 145 nm by using gate last process scheme. Top Si was thinned to ~8.5 nm. Dummy polysilicon gate was formed followed by a thin spacer (~8 nm). Faced raised source and drain were in situ epi-grown with boron doped. Silicon loss in source and drain areas should be carefully controlled to form high quality SiGe. RTA (rapid thermal annealing) was performed to drive in dopants to form extensions. After silicide and interlayer dielectric (ILD) formation, dummy gate was removed. Then, the preparations of interfacial layer and Hf-Ti-O higher *k* films for ETSOI PMOSFETS were entirely the same as those for the capacitor. TiN was selected as PMOSFET work function metal.

### Characterization of the ETSOI MOSFET Performance

The device performances were extracted from the typical transfer characteristics measurement of the drian current (*I*_*d*_) versus gate voltage (*V*_*g*_), where the threshold voltages (*V*_*t*_) were extracted through the constant current method when *I*_*d*_ equals to 0.1 μA (*W*/*L*).

## Results and Discussion

### Characterization of the Hf-Ti-O Higher *k*/IL/Si Stack

**Fig. 1:**
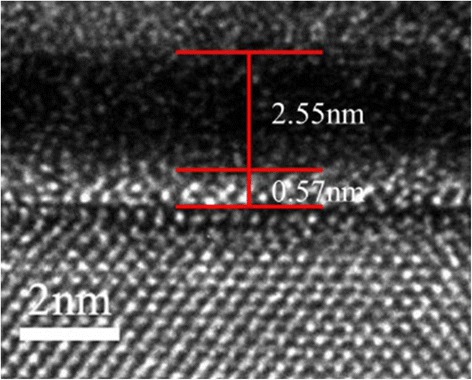
(Color online) High-resolution cross-sectional TEM image of the Hf-Ti-O/IL/Si stack

Figure 1 shows the high-resolution cross-section TEM image of Hf-Ti-O higher *k*/IL/Si stack. It could be seen that the Hf-Ti-O film is about 2.55 nm thick and remains amorphous after PDA at 450 °C. The interfacial layer thickness is about 0.57 nm.

**Fig. 2:**
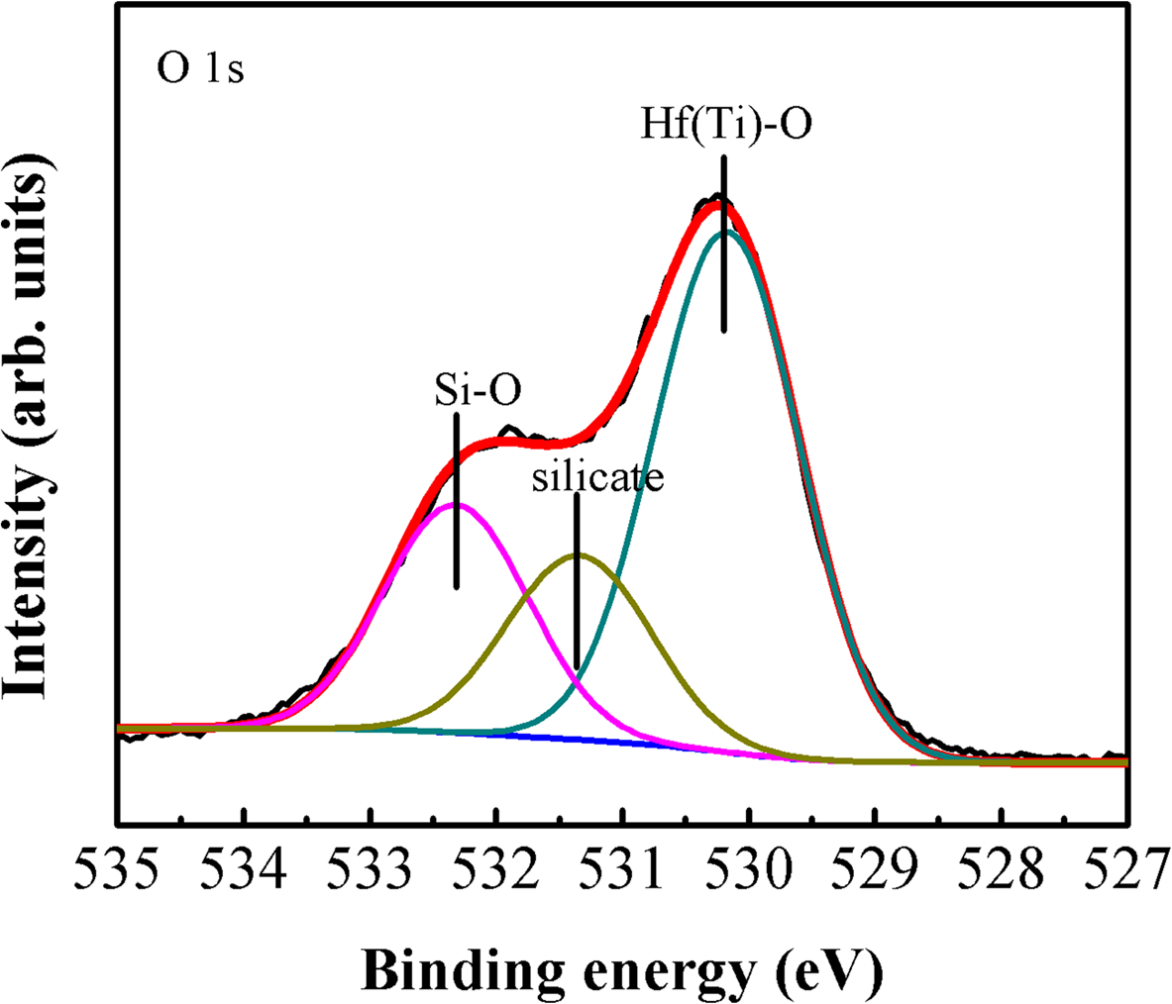
(Color online) XPS analysis of O 1s core level for Hf-Ti-O/IL/Si stack

Figure 2 illustrates the O 1s spectra of Hf-Ti-O/IL/Si stack. It is found that O 1s peak could be fitted by a standard Gaussian curve-fitting procedure and could be deconvoluted into three subpeaks, corresponding to Hf(Ti)-O (530.2 eV), Si-O (532.3 eV), and silicate Hf(Ti)-O-Si (531.4 eV), respectively, showing the formation of interfacial silicate. Additionally, XPS analysis shows that the atomic ratio of Ti/(Hf + Ti) is ~9.4%.

### Characterization of the Electrical Properties of Hf-Ti-O Higher k/IL/Si Stack

**Fig. 3:**
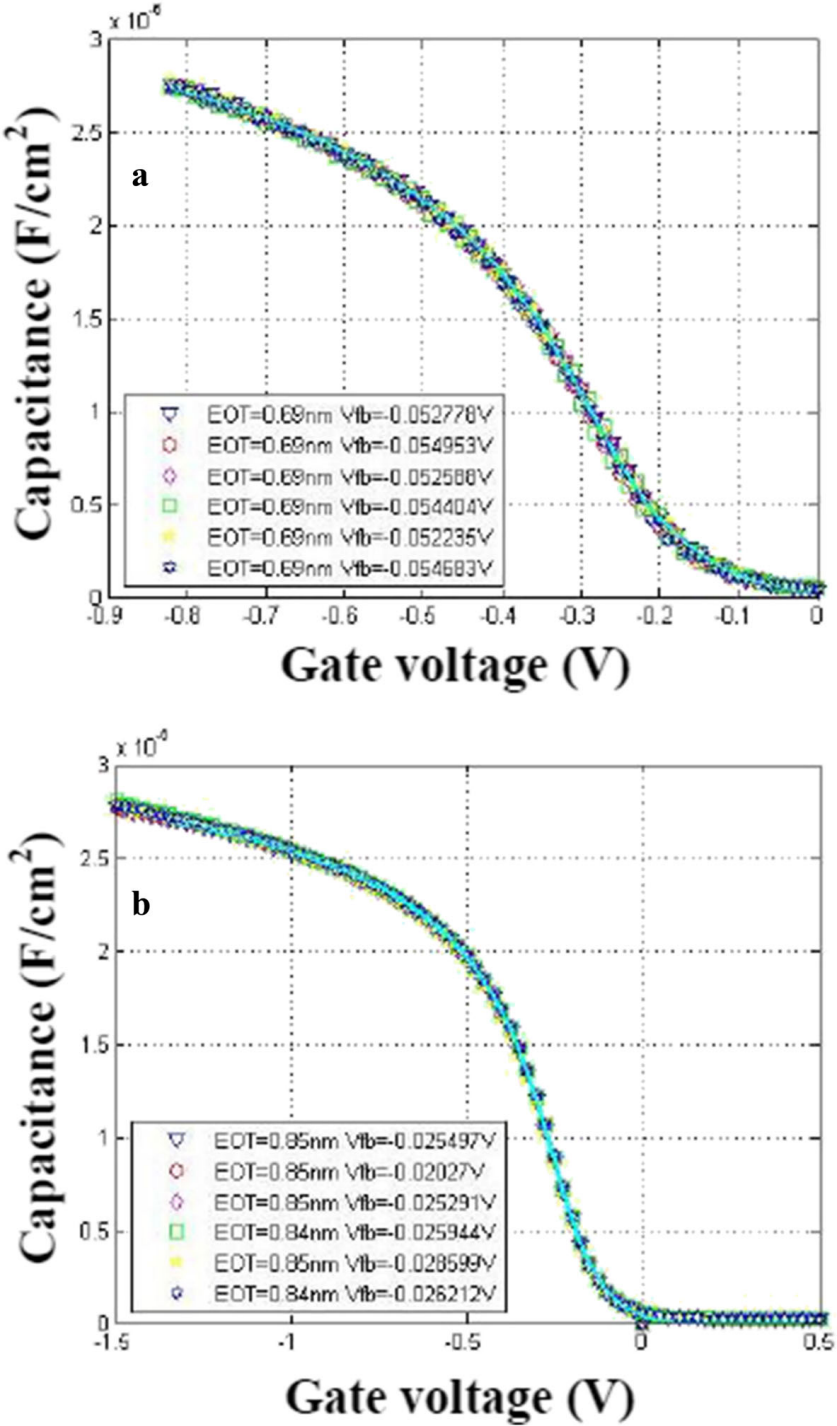
(Color online) Capacitance–voltage (*C*–*V*) curves at 1 MHz with a gate area of 100 × 100 μm^2^ **a** with Hf-Ti-O as gate dielectric and **b** with HfO_2_ as gate dielectric

Figure 3 demonstrates the measured and simulated *C*–*V* characteristics of the MOS capacitors with Hf-Ti-O (a) and HfO_2_ (b) as gate dielectrics, where the labeled dots denote the measured data and the solid curves show the simulated *C*–*V* curves. The EOT of TiN/Hf-Ti-O/IL/Si stack is extracted to be 0.69 nm from Fig. 3a. Furthermore, the effective permittivity of laminated Hf-Ti-O/IL (interfacial layer) is calculated to be as high as 17.6 for which two reasons are responsible. One is the higher permittivity of Hf-Ti-O higher *k* which should be greater than 30 on the basis of our previous study [15]. The other is the formation of interfacial silicate layer whose permittivity is greater than that of SiO_2_. In addition, the smooth and distortionless *C*–*V* curves also indicate the good interface quality and low interface state density. The flat-band voltage (*V*_fb_) is about −53.5 mV.

The extracted EOT of MOS capacitor with HfO_2_ as gate dielectric is 0.85 nm (as shown in Fig. 3b), greater than that of MOS capacitor with the same physical thickness Hf-Ti-O as gate dielectric. The calculated effective permittivity of laminated HfO_2_/IL is 14.3. Since the capacitors formed by the laminated high *k*/interfacial layer are series capacitors, the permittivities of HfO_2_ and Hf-Ti-O are calculated to be 20.2 and 30.0, respectively, while assuming the interfacial layer is of the same permittivity of ~6.2.

It is known that the low EOT is helpful in increasing the *I*_dsat_ (saturation drive current) [16] and reducing the short-channel effects (SCE) [17], thus improving the control capacity of gate bias voltage on the channel charges. Lower EOT could be obtained by using Hf-Ti-O higher *k* compared with HfO_2_ with the same physical thickness, suggesting Hf-Ti-O is beneficial to decrease SCE. Additionally, the extracted flat-band voltage (*V*_fb_) is about −25.1 mV.

**Fig. 4:**
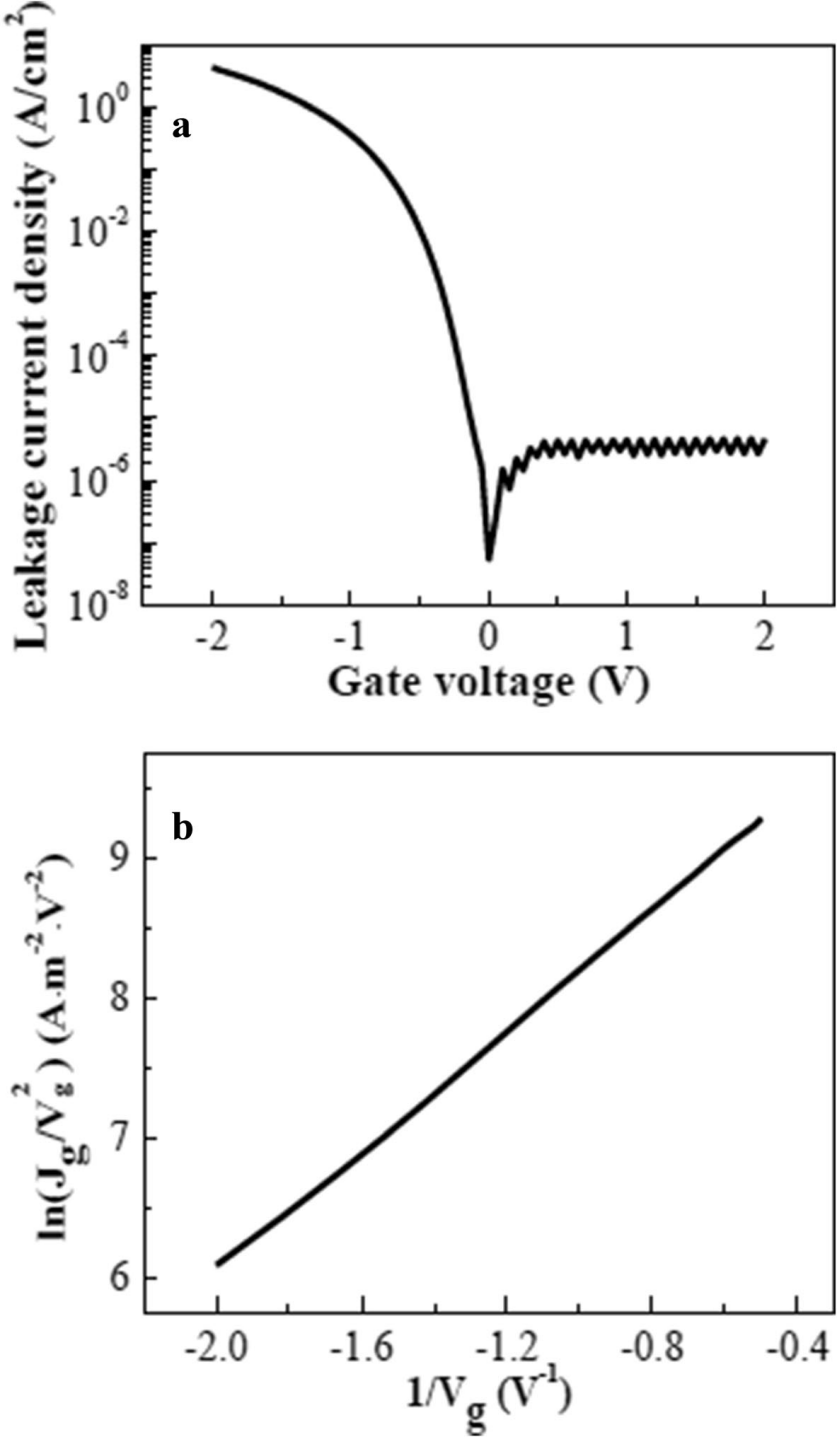
**a** Gate leakage current density versus gate voltage (*J*_*g*_–*V*_*g*_). **b** F-N tunneling mechanism

Integration of higher *k* materials, while limiting the fundamental increase in gate tunneling currents due to band-gap narrowing, are also challenges to be faced [1]. The gate leakage current density (*J*_*g*_) versus gate voltage (*V*_*g*_) for TiN/Hf-Ti-O/IL/Si stack is demonstrated in Fig. 4a. *J*_*g*_ < 1 A/cm^2^ @ (*V*_fb_ − 1)*V* is acceptable in 22-nm technology node and beyond. In the present study, the *J*_*g*_ at *V*_*g*_ = (*V*_fb_ − 1)*V* is 0.61 A/cm^2^, which is at least five orders lower than that of SiO_2_ at the same EOT of 0.69 nm [9, 18], and is slightly lower than that of TaN/TiO_2_/HfO_2_/Si stack with ~0.8-nm EOT [9], while the *J*_*g*_ at *V*_*g*_ = (*V*_fb_ − 1)*V* is 7.3 × 10^−2^ A/cm^2^ while using HfO_2_ as gate dielectric (not shown here).

It is known that TiO_2_ has smaller band gap and conduction band offset compared with HfO_2_ [16], leading to the reduction in band gap and conduction band offset of Hf-Ti-O. However, it is reported that if the Ti content in the Hf-Ti*-*O films is no higher than 21%, the conduction band offset is still greater than 1.06 eV [8]. Thus, the less Ti concentration of ~9.4% in Hf-Ti-O higher *k* influences the band gap, band offsets, and *J*_*g*_ not too much. In particular, the intentionally grown SiO_2_ interfacial layer also helps to decrease the gate leakage current. As a result, the acceptable gate leakage current density with low EOT of ~0.69 nm was obtained in this study.

It is known that oxygen vacancies are the intrinsic defects in HfO_2_ [19, 20]. As for TiO_2_, oxygen migration leads to oxygen vacancies [21], the common defects in TiO_2_. Oxygen vacancies decrease the resistivity of TiO_2_, which makes TiO_2_ an n-type semiconductor [22, 23]. Thus, the conduction mechanism through the Hf-Ti-O gate dielectric is expected to be dominated by the Poole–Frenkel emission, a trap-assisted mechanism due to oxygen vacancies. Whereas, it is found that in the gate voltage range of −0.5 to −2 V, there exists a relationship of $\ln \left(\frac{J_g}{V_g^2}\right)\propto \frac{1}{V_g},$ as shown in Fig. 4b, showing that the gate leakage current follows Fowler–Nordheim tunneling [17], an electric field-assisted tunneling mechanism. Fowler–Nordheim tunneling occurs when the electric field is rather large, namely the gate dielectric is rather thin. The possible suppression of oxygen vacancy formation or oxygen migration in the HfO_2_/TiO_2_/HfO_2_/IL stack still needs further study.

Low EOT of ~0.69 nm and acceptable gate leakage current density for the MOS capacitor indicate the scalability of Hf-based Hf-Ti-O higher *k* to 10-nm technology node and beyond.

### Characterization of the ETSOI MOSFET Performance

In our previous study, we found that for the ETSOI PMOSFET with a *W*/*L* of 3 μm/25 nm and with Hf-Ti-O as gate dielectric, when the linear threshold voltage (*V*_tlin_ at *V*_ds_ = −0.05 V) and saturation threshold voltage (*V*_tsat_ at *V*_ds_ = −0.9 V) were −0.21 and −0.16 V, respectively, the obtained *I*_on_/*I*_off_ ratio was 3.2 × 10^4^ [24], showing good performances while using Hf-Ti-O films as the high *k* gate dielectric.

**Fig. 5:**
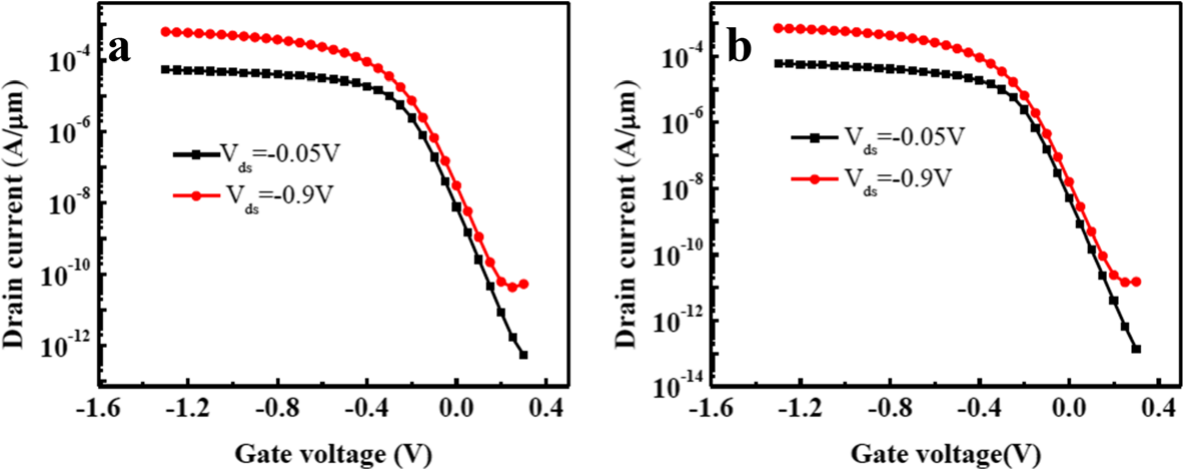
(Color online) Typical transfer characteristics (*I*_*d*_–*V*_*g*_) of two ETSOI PMOSFETs with *W*/*L* = 3 μm/25 nm (—*black square*—*V*_ds_ = −0.05 V,  *V*_ds_ = −0.9 V). **a** With HfO_2_ as gate dielectric. **b** With Hf-Ti-O as gate dielectric

For comparison, the ETSOI PMOSFET with the same physical thickness HfO_2_ as gate dielectric was prepared. Under the same process flow, the extracted *V*_tlin_ and *V*_tsat_ were −0.22 and −0.17 V, respectively, and the obtained *I*_on_/*I*_off_ ratio was 1.34 × 10^4^ (as shown in Fig. 5). In other words, under the same physical thickness, lower EOT and higher *I*_on_/*I*_off_ ratio could be obtained while utilizing Hf-Ti-O as gate dielectric, suggesting the potential of Hf-Ti-O as higher *k*.

**Fig. 6:**
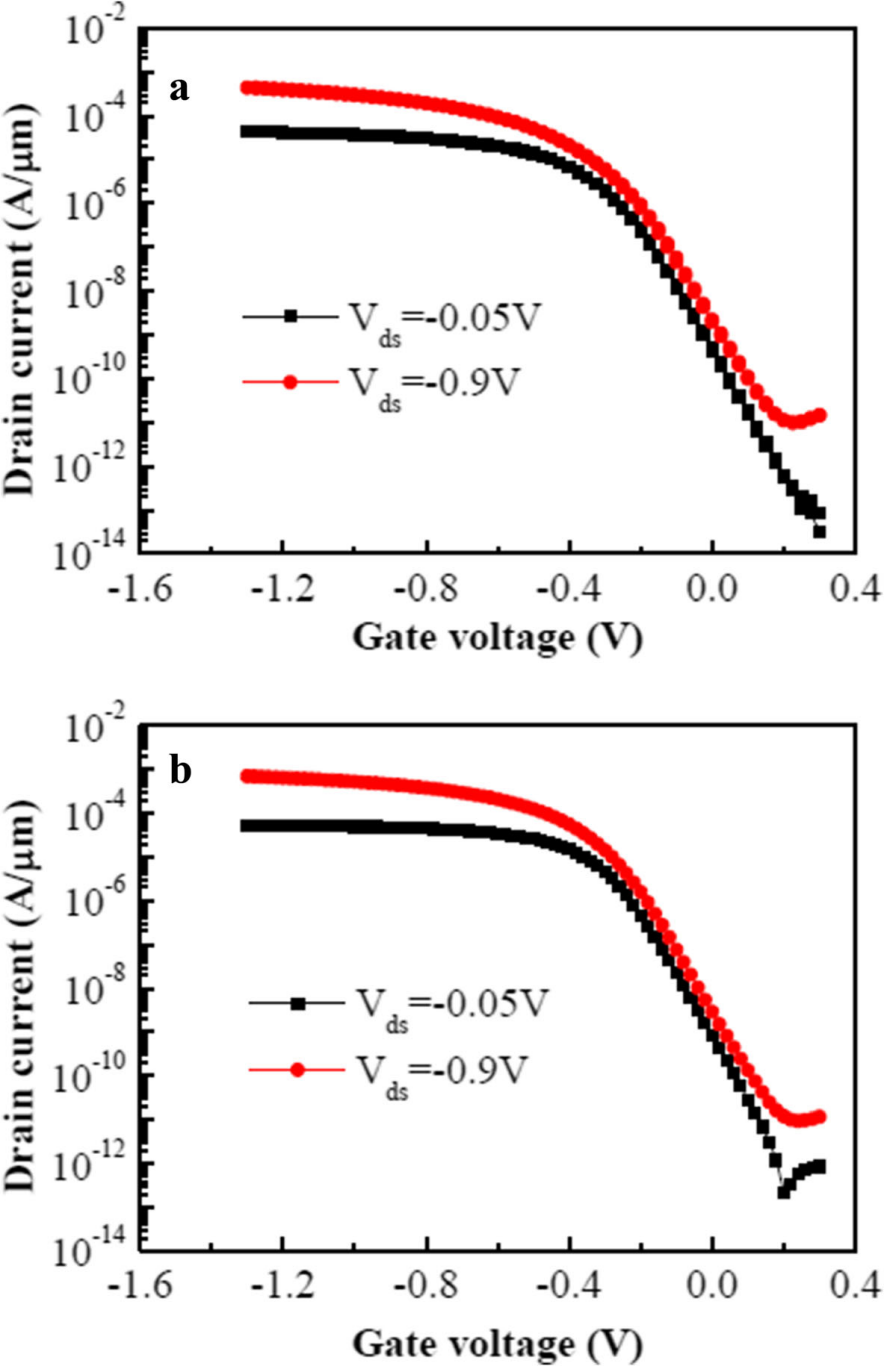
(Color online) Typical transfer characteristics (*I*_*d*_–*V*_*g*_) of two ETSOI PMOSFETs with Hf-Ti-O as gate dielectric ((—*black square*—*V*_ds_ = −0.05 V,  *V*_ds_ = −0.9 V). **a** *W*/*L* = 0.5 μm/25 nm. **b** *W*/*L* = 3 μm/40 nm

The *I*_on_/*I*_off_ ratio illustrates the switching performance of a MOSFET at a certain gate bias voltage. The higher the *I*_on_/*I*_off_ ratio, the shorter the switching time. In this study, some process parameters were adjusted in order to increase the *I*_on_/*I*_off_ ratios of ETSOI PMOSFETs with Hf-Ti-O as high *k* gate dielectric. Subsequently, two ETSOI PMOSFETs with two gate width/gate length of 0.5 μm/25 nm and 3 μm/40 nm were prepared. Figure 6 shows the typical transfer characteristics (*I*_*d*_–*V*_*g*_) of two ETSOI PMOSFETs. The device parameters are listed in Table 1. It is found that for both PMOSFETs, they have suitable threshold voltage in the range of −0.3~−0.2 V. For the PMOSFET with *W*/*L* of 0.5 μm/25 nm, the linear threshold voltage (*V*_tlin_ at *V*_ds_ = −0.05 V) and saturation threshold voltage (*V*_tsat_ at *V*_ds_ = −0.9 V) are −0.35 and −0.28 V, respectively. For the PMOSFET with *W*/*L* of 3 μm/40 nm, *V*_tlin_ and *V*_tsat_ are −0.27 and −0.22 V, respectively. For two PMOSFETs with *W*/*L* of 0.5 μm/25 nm and 3 μm/40 nm, their extracted on-state drive currents (*I*_on_) are 246 and 453 μA/μm, respectively, and their *I*_on_/*I*_off_ ratios are 1.12 × 10^5^ and 1.56 × 10^5^, respectively.

**Table 1:** Device parameters for ETSOI PMOSFETs with Hf-Ti-O as gate dielectric

Parameters	PMOSFET (*W*/*L* = 0.5 μm/25 nm)	PMOSFET (*W*/*L* = 3 μm/40 nm)
*I*_on_ (μA/μm)	246	453
*I*_off_ (A/μm)	2.2 × 10^−9^	2.9 × 10^−9^
*I*_on_/*I*_off_	1.12 × 10^5^	1.56 × 10^5^
*V*_tsat_ (V)	−0.28	−0.22
*V*_tlin_ (V)	−0.35	−0.27
*g*_*m*_ (μS/μm)	522	856
DIBL (mV/*V*)	82	59
SS (mV/decade)	70	66

Specially, ETSOI PMOSFETs with Hf-Ti-O as high *k* gate dielectric have superior short-channel control capacity with low DIBLs (DIBL, drain-induced barrier lowering) which are 82 and 59 mV/*V* for PMOSFETS with *W*/*L* of 0.5 μm/25 nm and 3 μm/40 nm, respectively. It is concluded that short-channel effects (SCE) are well controlled even for gate length downscaled to 25 nm.

Modern bulk MOSFETs usually have a subthreshold swing (SS) of 100 mV/decade or more, and typical values for the subthreshold swing in ETSOI MOSFETs are 70~80 mV/decade [25]. In this study, lower subthreshold swings, 70 and 66 mV/decade at *V*_ds_ = −0.9 V for PMOSFETs with a gate width/gate length of 0.5 μm/25 nm and 3 μm/40 nm, respectively, have been achieved. Moreover, low SS also indicates excellent interface quality [18].

In thin body devices, short-channel effects are controlled by the body thickness instead of the channel doping. The extremely thin top Si film limits naturally the source/drain junction depth as well as the depletion region of source/drain junction, thus improving the DIBL property related with short-channel effects and subthreshold characteristics, as well as lowering the static power consumption.

**Fig. 7:**
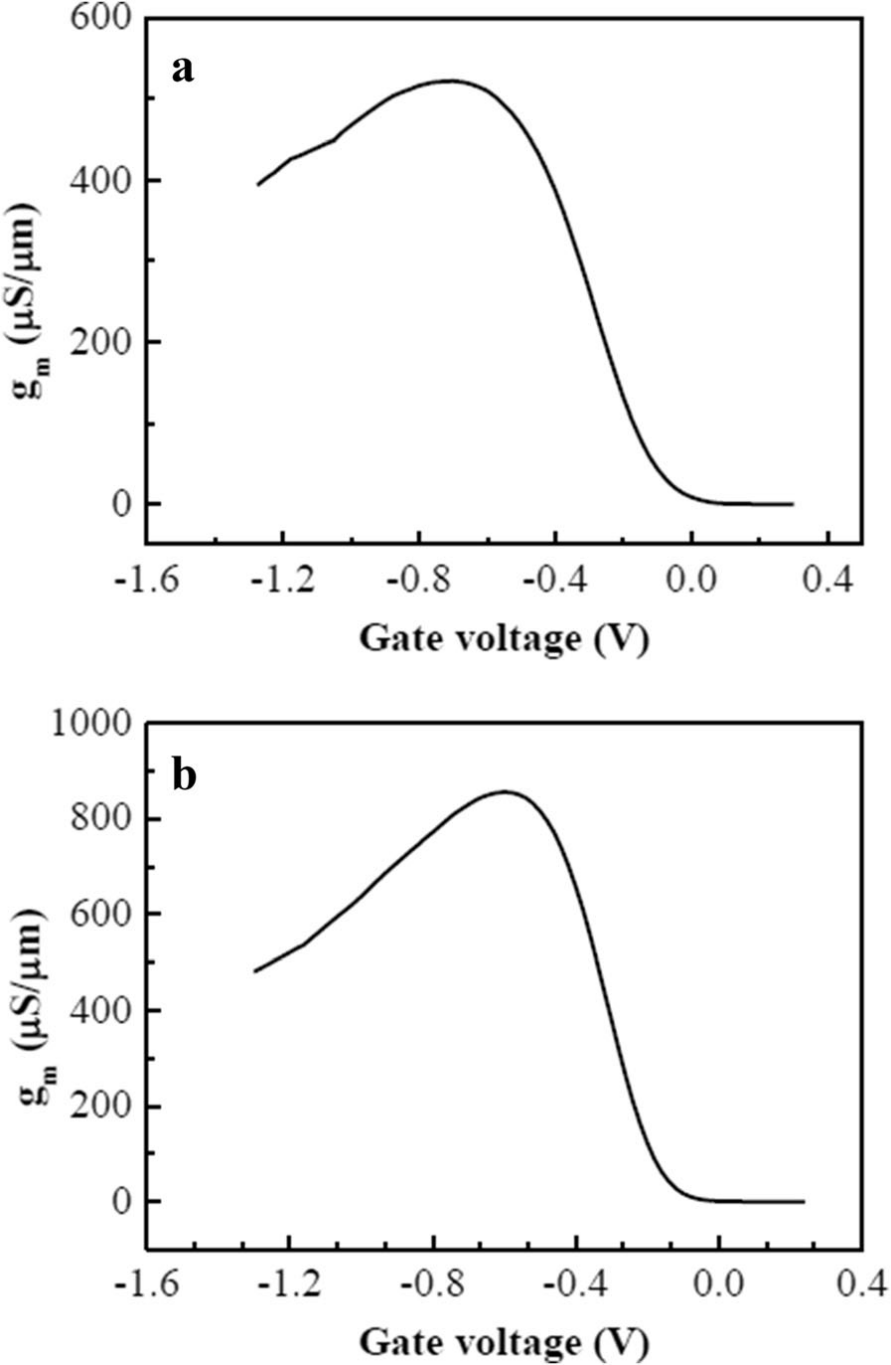
Transconductance (*g*_*m*_) versus gate voltage (*V*_*g*_) curves of the two ETSOI PMOSFETs. **a** *W*/*L* = 0.5 μm/25 nm. **b** *W*/*L* = 3 μm/40 nm

Figure 7 demonstrates the transconductance (*g*_*m*_) versus gate voltage (*V*_*g*_) curves. The high peak transconductances (*g*_*m*_) of 522 and 856 μS/μm (also listed in Table 1) for PMOSFETs with *W*/*L* of 0.5 μm/25 nm and 3 μm/40 nm, respectively, also show well-behaved transistor characteristics.

## Conclusions

In summary, low EOT of ~0.69 nm, acceptable gate leakage current density, and good PMOSFET performances including high I_on_, *I*_on_/*I*_off_ ratio, *g*_*m*_, and suitable threshold voltage, as well as low *I*_off_, DIBL, and SS for two ETSOI PMOSFETs with a gate width/gate length of 0.5 μm/25 nm and 3 μm/25 nm could be obtained while utilizing Hf-Ti-O higher *k* gate dielectric, appropriate high *k*/Si interface processing technology, and metal gates. The conduction mechanism through the gate dielectric in NMOS capacitor is dominated by the F-N tunneling in the gate voltage range of −0.5 to −2 V instead of Poole–Frenkel emission. Compared with HfO_2_, lower EOT and better ETSOI PMOSFET performance could be obtained while using Hf-Ti-O gate dielectric. Namely, Hf-Ti-O has the potentiality to be used as higher *k* and is promising in extending the application of Hf-based high *k* in 10-nm technology node and beyond, although further research on optimizing technological parameters to improve the performances of ETSOI PMOSMETs is still needed. The combination of higher *k* gate dielectric material and new ETSOI device structure will help to improve transistor performance and continue CMOS scaling.

## References

[1] Semiconductor Industry Association. National technology roadmap for semiconductors. ITRS 2013, ITRS 2009, http://www.itrs2.net/

[2] Mistry K, Allen C, Auth C, Beattie B (2007). A 45nm logic technology with high-k + metal gate transistors, strained silicon, 9 Cu interconnect layers, 193 nm dry patterning, and 100% Pb-free packaging.

[3] Ando T (2012). Ultimate scaling of high-k gate dielectrics: higher-k or interfacial layer scavenging?. Materials.

[4] Frank MM (2011). Proceedings of the European solid-state circuits conference.

[5] Li M, Zhang Z, Campbell SA, Gladfelter WL (2005). Electrical and material characterizations of high-permittivity Hf_x_Ti_1-x_O_2_ gate insulators. J Appl Phys.

[6] Lee C, Ghosez P, Gonze X (1994). Lattice dynamics and dielectric properties of incipient ferroelectric TiO_2_ rutile. Phys Rev B.

[7] Ramani K, Singh RK, Cracium V (2008). Hf–O–N and HfO_2_ barrier layers for Hf–Ti–O gate dielectric thin films. Microelectron Eng.

[8] Ye C, Wang H, Zhang J, Ye Y, Wang Y (2010). Composition dependence of band alignment and dielectric constant for Hf_1−x_Ti_x_O_2_ thin films on Si(100). J Appl Phys.

[9] Rhee SJ, Kang CS, Choi CH, Kang CY et al (2004) Improved electrical and material characteristics of hafnium titanate multi-metal oxide n-MOSFETs with ultra-thin EOT (∼8 Å) gate dielectric application. Electron Devices Meeting, 2004. IEDM Technical Digest. IEEE International (IEEE, 2004), New York, pp. 837–840

[10] Cheng K, Khakifirooz A, Kulkarni P, Ponoth S (2010). Extremely thin SOI (ETSOI) technology: past, present, and future. Proceedings of 2010 IEEE International SOI Conference.

[11] Khakifirooz A, Cheng K, Kulkarni P, Cai J (2010). Challenges and opportunities of extremely thin SOI (ETSOI) CMOS technology for future low power and general purpose system-on-chip applications. 2010 Int. Symp. On VLSI Technology Systems and Applications (VLSI-TSA).

[12] Faynot O, Andrieu F, Weber O, Fenouillet-Béranger C et al (2010) Planar fully depleted soi technology: a powerful architecture for the 20 nm node and beyond. Electron Devices Meeting (IEDM), 2010 IEEE International IEDM Tech. Dig., 2010, pp. 3.2.1–3.2.4. doi:10.1109/IEDM.2010.5703287

[13] Cheng K, Khakifirooz A, Kulkarni P, Ponoth S (2011). ETSOI CMOS for system-on-chip applications featuring 22 nm gate length, sub-100 nm gate pitch, and 0.08 μm^2^ SRAM cell. 2011 Symposium on VLSI Technology (VLSIT).

[14] Khakifirooz A, Cheng K, Reznicek A, Adam T (2012). Scalability of extremely thin SOI (ETSOI) MOSFETs to sub-20-nm gate length. IEEE Electron Device Lett.

[15] Chen XQ, Xiong YH, Wei F, Zhao HB et al (2015) study of electrical behavior of Hf-Ti-O higher-k dielectric for ETSOI MOSFET application. International Symposium on Material, Energy and Environment Engineering (ISM3E 2015), part of series: Advances in Engineering Research. pp 587–590 doi:10.2991/ism3e-15.2015.142

[16] Wilk GD, Wallace RM, Anthony JM (2001). High-κ gate dielectrics: current status and materials properties considerations. J Appl Phys.

[17] Sze SM, Ng KK (2007). Physics of semiconductor devices.

[18] Lu N, Li HJ, Gardner M, Kwong DL (2005). Higher *k* HfTaTiO gate dielectric with improved material and electrical characteristics. Device Research Conference Digest, 2005. DRC ‘05.

[19] Xiong K, Robertson J, Gibson MC, Clark SJ (2005). Defect energy levels in HfO2 high-dielectric-constant gate oxide. Appl Phys Lett.

[20] Lucovsky G, Hinkle CL, Fulton CC, Stoute NA (2006). Intrinsic nanocrystalline grain-boundary and oxygen atom vacancy defects in ZrO_2_ and HfO_2_. Radiat Phys Chem.

[21] Brown SL, Rossnagel SM, Bruley J, Copel M (2010). Oxygen migration in TiO_2_-based higher-k gate stacks. J Appl Phys.

[22] Yagi E, Hasiguti RR, Aono M (1996). Electronic conduction above 4K of slightly reduced oxygen-deficient rutile TiO_2-x_. Phys Rev B.

[23] Frank MM, Kim SB, Brown SL, Bruley J (2009). Scaling the MOSFET gate dielectric: from high-k to higher-k?. Microelectron Eng.

[24] Chen XQ, Zhao HB, Xiong YH, Wei F (2016). Study of Hf-Ti-O thin film as high-k gate dielectric and application for ETSOI MOSFETs. J Electron Mater.

[25] Khakifirooz A, Cheng K, Liu Q, Nagumo T (2012). Extremely thin SOI for system-on-chip applications. Proceedings of the IEEE 2012 Custom Integrated Circuits Conference, 2012 IEEE.

